# Co-Occurring Risk Factors for Alcohol Dependence and Habitual Smoking: Update on Findings From the Collaborative Study on the Genetics of Alcoholism

**Published:** 2006

**Authors:** Richard A. Grucza, Laura J. Bierut

**Affiliations:** Richard A. Grucza, Ph.D., M.P.E., is an assistant professor and Laura J. Bierut, M.D., is an associate professor in the Department of Psychiatry, Washington University School of Medicine, St. Louis, Missouri

**Keywords:** Alcohol and tobacco, alcohol, nicotine, co-use, co-abuse, biological basis, animal studies, animal models, genetics, selected strains, mesolimbic dopamine system, cross-tolerance

## Abstract

Habitual smoking and alcohol dependence frequently co-occur, and the genetic factors that influence both conditions appear to overlap. The Collaborative Study on the Genetics of Alcoholism (COGA) has investigated genetic factors that contribute to both alcohol dependence and habitual smoking. Using a sample of families densely affected with alcohol dependence, COGA investigators have identified regions of the genome likely to contain genes that specifically contribute to alcohol dependence and habitual smoking, as well as regions likely to contain genes that contribute to the development of both conditions. Further genetic analyses (i.e., candidate gene studies) have helped identify specific genes that may contribute to the development of alcohol dependence and habitual smoking. These analyses have implicated several genes that encode parts of receptors for the neurotransmitter gamma-aminobutyric acid (GABA) in the development of alcohol or nicotine dependence, respectively. Other studies have identified additional candidate genes for alcohol or nicotine dependence. The results to date suggest that both common and drug-specific genetic influences play a role in the development of alcohol and nicotine dependence.

Excessive alcohol use and smoking are among the top contributors to preventable early mortality in the United States today (Mokdad et al. 2004). Alcohol-related deaths stem from a variety of chronic diseases, including liver disease, cancers, and cardiovascular disease, as well as from acute consequences of alcohol consumption, such as alcohol-related car crashes, other accidents, suicides, and acute alcohol toxicity. Moreover, the public health impact of heavy drinking and alcohol dependence is not limited to effects on the individual drinker. Factors such as alcohol-related car crashes and violence as well as harm to the unborn children of pregnant women who drink (e.g., fetal alcohol syndrome) also contribute to the societal cost of alcohol problems (Harwood 2000). Smoking similarly is associated with significant mortality and morbidity caused by multiple cancers, lung disease, and heart disease.

More people are addicted to nicotine (the active ingredient in tobacco) and alcohol than to any other drugs of abuse (Grant et al. 2004). Moreover, alcohol and nicotine dependence often occur together in the same person, suggesting that the two disorders are not independent. Thus, the prevalence of nicotine dependence among people with alcohol dependence is more than three times higher (45.4 percent) than in the general population (12.8 percent). Likewise, the prevalence of alcohol dependence is about four times higher among people with nicotine dependence (13.5 percent) than in the general population (3.8 percent) (Grant et al. 2004).

Although environmental factors (e.g., availability of the drugs) have a substantial impact on the development of alcohol and nicotine dependence, it is well established that genetic factors also play a large role. Studies of identical and fraternal twins have consistently determined that the heritability of alcohol dependence—the estimated degree of variability in the risk of alcohol dependence that is accounted for by genetic factors—is between 50 and 60 percent; the heritability of nicotine dependence is comparable (for a review, see Tyndale 2003). Furthermore, twin-based research has indicated that the genetic factors that contribute to alcohol and nicotine dependence may overlap. In those studies, the genetic correlation between the two disorders—which estimates the degree of overlap in genetic factors and can range between 0 (no overlap) and 1 (complete overlap)—was found to be 0.68, indicating substantial overlap (True et al. 1999). Thus, in addition to the genetic factors that are specific for each disorder, certain genetic factors increase the risk of developing both alcohol and nicotine dependence.

This review presents recent results from the Collaborative Study on the Genetics of Alcoholism (COGA) regarding the genetic factors influencing alcohol dependence and habitual smoking. After providing an overview of design, methods, and findings of COGA, the article also briefly describes how advances in genetic technology have led to compelling new findings in the search for specific genes related to alcohol and nicotine dependence. This discussion highlights recent findings on specific genes and gene variants that have been identified as risk factors for one or both of these disorders.

## Overview of COGA

COGA is an ongoing, comprehensive family and genetic study of alcohol dependence and related disorders that is conducted at multiple sites in the United States. In addition to studying genetic and other biological factors related to alcohol use, abuse, and dependence, the investigators have collected data on a broad variety of behavioral variables, psychiatric disorders, personality traits, family history, and many other risk factors.

The study recruited patients seeking treatment for alcoholism; these participants are referred to as probands. Subsequently, the study also recruited the probands’ family members for further evaluation. The assessment of these families took place in two stages. During the first stage, both the probands and their family members completed extensive interviews that allowed the investigators to determine whether participants met the diagnostic criteria for alcohol abuse or dependence. To be classified as alcohol dependent, participants had to meet both the diagnostic criteria for alcohol dependence listed in the *Diagnostic and Statistical Manual of Mental Disorders, Third Edition, Revised* (American Psychiatric Association [APA] 1987) and the Feighner definite criteria for alcoholism (Feighner et al. 1972). Other behaviors, including smoking behaviors, also were assessed during the first stage. This allowed researchers to examine the relationship between alcohol dependence and smoking among family members.

If a proband was found to have at least two siblings with alcohol dependence, the family was recruited for the second stage of the study. During this stage, blood samples were drawn to collect DNA for genetic studies aimed at identifying genes that contribute to the risk of alcohol dependence. Because smoking behaviors also had been assessed during the first stage, the DNA samples also could be analyzed for genes that might contribute to both smoking and alcohol dependence.

It is important to note that in the studies described here, the researchers only studied habitual smoking—defined as smoking 20 cigarettes (i.e., one pack) or more daily for at least 6 months at some time in life—but did not determine whether the participants were dependent on nicotine. A later study involving a subset of the participants, however, included an interview that assessed nicotine dependence. In that study, investigators determined that 71 percent of the habitual smokers were nicotine dependent (Bierut et al. 2004). The identification of potential genetic differences between habitual smokers with and without nicotine dependence constitutes an interesting topic for future analyses.

COGA researchers used several approaches to determine the influence of genetic factors on the development of alcohol dependence and/or habitual smoking, including family studies, genetic linkage analyses, and candidate gene association studies. These approaches and their main findings are reviewed in the following sections.

## Family Studies of Alcohol Dependence and Habitual Smoking

When trying to assess the importance of genetic influences in the development of a disorder, the first step usually is to determine whether the disorder aggregates in families. Likewise, the co-aggregation of two disorders within families can provide evidence that common genetic factors influence both conditions. Using data from more than 1,000 families participating in COGA, Bierut and colleagues (1998, 2000) examined the transmission of both alcohol dependence and habitual smoking within families and determined risk factors for both conditions. This analysis found that the greatest risk factor for developing dependence on a drug is being already dependent on another drug.

The level of comorbidity between alcohol dependence and smoking was particularly high, and both alcohol dependence and habitual smoking clustered in families. Specifically, the investigators found that siblings of an alcohol-dependent proband were at 1.7 times higher risk of becoming habitual smokers than were siblings of people who were not dependent on alcohol. In addition, habitual smoking in alcohol-dependent probands further increased their siblings’ risk of becoming habitual smokers by a factor of 1.8 (Bierut et al. 1998, 2000). These findings provide further evidence of both common and drug-specific influences in the development of alcohol dependence and habitual smoking.

## Genetic Linkage Analysis of Habitual Smoking and Alcohol Dependence

Twin studies and other family studies such as the ones described above have suggested that genetic factors impact both alcohol dependence and smoking. To extend the family studies of these disorders and systematically identify genetic regions that contribute to alcohol dependence, habitual smoking, or both, COGA researchers used a method known as genetic linkage analysis. This approach involves searching for DNA sequence variations throughout the genome, using DNA sequences (i.e., markers) whose location on the chromosomes is known. Hundreds of markers have been identified in recent years that cover all human chromosomes. In many cases, several variants, or alleles, exist for a given marker, so researchers can determine not only whether the marker is present or absent in family members but also whether family members share a particular marker allele or not.

When relatives who resemble each other in terms of a disease or other characteristics (i.e., phenotype^1^) share a certain genetic marker at a rate greater than expected based on chance alone, this strongly suggests that a gene located near the marker influences the disease. For example, two siblings share, on average, 50 percent of their genes and are therefore also expected to share a given marker 50 percent of the time. If both siblings have the same disorder, however, they should share those regions of the genome that contribute to the development of the disorder more than 50 percent of the time.

Because genetic factors had been implicated in the development of both alcoholism and smoking, and because some of these genetic factors were expected to overlap, COGA investigators performed a genomic screen for both disorders in families of alcoholics (Bierut et al, 2004). In these analyses, the degree of sharing between siblings was examined for 336 genetic markers from throughout the genome. The genomic screen identified several chromosomal regions that may contain genes influencing the development of habitual smoking but not of alcohol dependence. For example, two regions on chromosome 9 exhibited increased allele sharing among sibling pairs who were both habitual smokers; therefore, these regions may play a role in the development of this disorder. However, these regions showed no significant evidence of a genetic contribution to alcohol dependence—that is, they were not shared at rates greater than chance among pairs of alcohol-dependent siblings. Therefore, these regions likely contain genes that specifically influence the susceptibility to habitual smoking.

Other DNA regions were implicated in the development of alcohol dependence but not habitual smoking. For instance, one region on chromosome 7 appeared to be linked to alcohol dependence but showed no evidence of being linked to habitual smoking (Bierut et al. 2004).

Some of the strongest findings involved chromosomal regions—for example, on chromosome 2—that appear to contribute to both habitual smoking and alcohol dependence. The degree of allele sharing was highest when both disorders were evaluated simultaneously— that is, the strongest evidence for increased sharing was seen among sibling pairs with both alcohol dependence and habitual smoking. Additional analyses indicated that a region on chromosome 3 may contribute to both conditions (Bierut et al. 2004). Hence, linkage findings support the hypothesis that certain genes contribute to both alcoholism and habitual smoking.

**Table:** Summary of Findings of Linkage Analyses and Candidate Gene Association Studies of Chromosome Regions and Genes That May Predispose to Alcohol Dependence, Nicotine Dependence, or Both

Condition Studies	Chromosome Regions Identified Through Linkage Analysis	Candidate Genes Identified Through Association
Alcohol dependence only	Chromosome 4	GABRA2 gene (component of the GABA_A_ receptor)
	Chromosome 7	CHRM2 gene (component of the muscarinic M2 receptor*)
Nicotine dependence only	Chromosome 9	GABAB2 gene (component of the GABA_B_ receptor)
Both alcohol and nicotine dependence	Chromosome 2	?
	Chromosome 3	?

* This gene also may contribute to the risk of depression.

NOTE: GABA = gamma-aminobutyric acid.

In summary, COGA was the first study to screen the genome for regions involved in both alcohol dependence and habitual smoking. The findings provide evidence that some chromosomal regions are linked to both disorders, whereas other regions are specific to one of the disorders (see Table). Such linkage studies, however, can only examine markers that signal the proximity of genes contributing to a given disorder. Additional studies have already identified specific candidate genes that may contribute to alcohol and/or nicotine dependence. Such candidate gene studies are described in the following section.

## Candidate Gene Association Studies for Alcohol and Nicotine Dependence

Once linkage analyses have identified chromosome regions contributing to alcohol dependence, the next step is to examine individual candidate genes that are known to be located in those regions. This is typically done using gene association studies. In contrast to linkage studies, which examine whether relatives with a disorder share a given marker more often than expected by chance, association studies consider whether a particular variant of a gene is found more often in people with the disorder than in people without the disorder. Whereas linkage studies identify relatively large chromosomal regions that can contain numerous genes as contributors to the development of the disorder, association studies detect significant effects over smaller DNA regions and can be used to identify specific genes which contribute to the development of the disorder. Moreover, whereas linkage analyses must be conducted with relatives, association studies can be performed using either families or unrelated probands and controls.

Association studies assess polymorphisms— genetic differences between individuals at specific locations of the genome. These polymorphisms, which can be located within a gene (where they would give rise to different gene variants, or alleles) but also outside of genes, are used to identify regions of the genome that are located at or near a disease susceptibility gene. Most recently, candidate gene association studies have been conducted using single-nucleotide polymorphisms (SNPs). Nucleotides are the building blocks of the DNA. Accordingly, SNPs are DNA segments that differ in only a single building block and can therefore be considered the smallest unit of human genetic variation. The identification of SNPs became possible only after the Human Genome Project, which deciphered the entire human DNA sequence, had been completed, and additional technological advances since then have facilitated the study of SNPs in large samples. The main advantage of using SNPs in candidate gene studies is that they allow researchers to locate disease susceptibility genes with greater precision than before. Although earlier genetic studies had to rely on a relatively small number of polymorphic markers, the density of markers has dramatically increased thanks to the SNPs.

Using SNPs, COGA researchers have studied several candidate genes that may contribute to the development of alcohol and/or nicotine dependence, most prominently genes encoding parts of the receptors through which the brain chemical (i.e., neurotransmitter) gamma-aminobutyric acid (GABA) acts on its target cells.

### GABA Receptor Genes

GABA is a neurotransmitter—a brain chemical that is released by nerve cells (neurons) to transmit signals to neighboring neurons. GABA is a major inhibitory neurotransmitter in the nervous system. This means that GABA acts on its target neurons to prevent other nerve signals from being propagated across those neurons. To exert this inhibitory effect, GABA interacts with complex protein molecules (i.e., receptors) that are embedded in the membrane of the signal-receiving neurons. Several different types of GABA receptors exist, such as the GABA_A_ and GABA_B_ receptors. Each of these receptors, in turn, is comprised of several proteins that are encoded by a corresponding number of genes. For example, genes called *GABRA2, GABRA4, GABRB1,* and *GABRG1* code for components of the GABA_A_ receptor, and a gene called *GABAB2* codes for one component of the GABA_B_ receptor. Researchers previously had demonstrated that alcohol directly interacts with the GABA_A_ receptor, enhancing the inhibitory effects of GABA and resulting in alcohol’s sedative effect (Davies 2003).

Consistent with functional studies of the GABA_A_ receptor, COGA linkage studies had identified a large DNA region located on chromosome 4, which contains the *GABRA2, GABRA4, GABRB1,* and *GABRG1* genes, as being related to the risk of alcoholism (Porjesz et al. 2002). For these analyses, COGA investigators used a strategy that tests for endophenotypes—basic biologic features of complex disorders—rather than the usual phenotype (e.g., alcoholism). In this case, the endophenotype was an electrophysiological measure of brain function known as the P300 component of an event-related potential (ERP). ERPs are specific brain waves that occur in response to a sudden stimulus (e.g., a sudden sound or light). Within these brain waves, a particular spike typically occurs about 300 milliseconds after the stimulus; this is the P300 wave. A reduced size (i.e., reduced amplitude) or delayed appearance of the P300 brain wave has been found both in alcohol-dependent people and in people at increased genetic risk of becoming alcohol dependent (Porjesz et al. 2003). Hence, both linkage results and molecular biological studies have suggested that variation in the GABA_A_ receptor might be involved in alcohol dependence.

To evaluate this hypothesis, COGA researchers systematically selected and characterized (i.e., determined the exact nucleotide sequence of) a series of SNPs from GABA_A_ receptor genes located on chromosome 4. The investigators identified several SNPs located in the *GABRA2* gene that were associated with alcohol dependence; in the neighboring genes encoding other GABA_A_ receptor components, however, no SNPs related to alcohol dependence were identified (Edenberg et al. 2004). Other researchers have replicated this finding in several independent samples (Covault et al. 2004; Lappalainen et al. 2005; Fehr et al. 2006). These findings indicate that the *GABRA2* gene contributes to the development of alcohol dependence.

Linkage studies aiming to identify chromosome regions associated with nicotine dependence also have been followed by successful candidate gene studies. Several linkage studies have implicated a region on chromosome 9, which includes genes for the GABA_B_ receptor, in the development of nicotine dependence (Li et al. 2003; Bierut et al. 2004; Gelernter et al. 2004). Based on these linkage analyses, Li (2006) conducted a candidate gene association study of the *GABAB2* gene, which lies within the area of chromosome 9 and encodes a component of the GABA_B_ receptor. Several SNPs in this gene were associated with nicotine dependence.

Thus, it appears that GABA receptors play a role in the development of both alcohol and nicotine dependence. It is known that GABA modulates the rewarding effects of drugs of abuse; accordingly, nicotine appears to enhance the rewarding effects of other drugs by interfering with GABA-mediated inhibition of such signals. Consistent with this hypothesis, animal studies suggest that the GABA_B_ receptor is a potential target for pharmacological treatment of nicotine dependence as well as dependence on other drugs (Cousins et al. 2002). However, the exact role of the GABA receptors in the development of dependence appears to differ for alcohol and nicotine. Whereas alcohol acts directly on the GABA_A_ receptor, the effect of nicotine on GABA_B_ function appears to be indirect.

Hence, these genetic analyses suggest that variations in GABA receptors are involved in both alcohol dependence and nicotine dependence. As described earlier, GABA, as an inhibitory neurotransmitter, prevents nerve signals from being propagated across neurons. Accordingly, dysfunction in GABA receptors may lead to excess activation of neurons (i.e., central nervous system hyperexcitability)—an assumption that is consistent with the observed contribution of differences in the *GABRA2* gene to variability in the P300 brain waves described above. Some authors have suggested that these electrophysiological differences may be associated not only with alcohol dependence but with a broader spectrum of drug dependence, including nicotine dependence, and other behavioral disorders (Iacono et al. 2002).

In summary, variations in the *GABRA2* gene are associated with differences in electrophysiological measures (e.g., the P300 wave) that are indicative of vulnerability to a variety of addictions. In addition, several association studies have implicated *GABRA2* in alcohol dependence. Finally, the findings of one association study and several suggestive linkage studies have implicated variations in the *GABAB2* gene in nicotine dependence. Because *GABRA2* and *GABAB2* have similar functions, it is possible that these and other genes encoding GABA receptor components may contribute to a variety of addictive disorders. Furthermore, the role of *GABRA2* in determining P300 brain waves suggests that electrophysiological studies may help to identify and elucidate the function of variants within this family of genes that are associated with an increased risk of dependence on alcohol and other drugs.

### Other Candidate Genes

Although the studies reviewed in detail here have focused on the association between the GABA system and alcohol and nicotine dependence, other genes associated with these disorders also have been identified. These candidate genes fall into two broad classes: one group of genes has been identified in studies of nicotine or alcohol dependence but plays important roles throughout the central nervous system and is therefore likely associated with other addictions or psychiatric disorders as well. The second group of genes is specifically related to the actions of alcohol or nicotine and therefore may be specific to a given disorder.

#### Genes Potentially Associated with Multiple Disorders

Linkage analyses by COGA investigators suggested that a region on chromosome 7 also was associated with the risk for alcoholism. Closer examination of this broad linkage region identified a candidate gene called *CHRM2* (Wang et al. 2004). This gene encodes another neurotransmitter receptor called the muscarinic M2 receptor, which interacts with the neurotransmitter acetylcholine.^2^ Nerve cell systems using acetylcholine as a neurotransmitter (i.e., cholinergic systems) have long been implicated in depression. For example, compounds that mimic the effects of acetylcholine (i.e., cholinergic agonists) can induce a depressed mood in animals and humans (Bymaster and Felder 2002). In mice, the muscarinic M2 receptor also appears to be involved in mediating certain stress responses (Hemrick-Luecke et al. 2002).

Linkage analyses demonstrated that the region containing the *CHRM2* gene contributed to electrophysiological differences as well as to the risk of alcohol dependence and major depression. (Jones et al, 2004). Subsequent association studies both in the COGA sample and in an independent sample identified several SNPs in the *CHRM2* gene that were associated with alcohol dependence, major depression, and comorbid alcoholism and depression, as well as with electrophysiologic measures (Wang et al. 2004; Luo et al. 2005). Thus, the *CHRM2* gene appears to contribute to multiple alcohol-related phenotypes.

Other genes involved in important nerve cell signaling pathways, such as those using the neurotransmitters acetylcholine, dopamine, serotonin, and endogenous opioids, have been found to be associated with alcohol dependence, nicotine dependence, or both (for reviews see Dick and Foroud 2003; Tyndale 2003; Dick and Bierut 2006; Li 2006). For example, several different types of nicotinic receptors have been implicated in the development of nicotine dependence (Li 2006). Because these receptors serve a number of important neurobiologic functions, they constitute candidate genes for other disorders as well.

#### Genes Specific for Alcohol or Nicotine Dependence

Variants of genes involved in the breakdown (i.e., metabolism) of alcohol are among the most commonly studied genetic risk or protective factors for alcoholism. In the body, alcohol is first converted to acetaldehyde by alcohol dehydrogenase (ADH) enzymes; subsequently, acetaldehyde is converted to acetate by aldehyde dehydrogenase (ALDH) enzymes. Genetic studies have repeatedly implicated members of the gene families encoding these enzymes as influencing a person’s risk of becoming alcohol dependent (for recent reviews, see Tyndale 2003; Dick and Bierut 2006; Edenberg et al. 2006).

Likewise, genes involved in nicotine metabolism—for example, genes encoding a family of enzymes known as cytochrome P450—have been suggested to play a role in nicotine dependence and smoking-related behavior (Tyndale 2003). Thus, genes specifically involved in the metabolism of a drug may contribute to vulnerability to dependence on that drug but not on other drugs.

## Conclusion

In recent years, technological advances have vastly increased researchers’ ability to precisely locate genes contributing to disease susceptibility. For example, studies that incorporate advances in SNP identification technology into genome-scanning methods, which will allow researchers to examine many more locations in the genome than previous approaches, currently are underway. Improved precision when mapping genes will result in more robust and reproducible findings.

A wealth of data suggests that common genetic pathways are involved in the development of alcohol and nicotine dependence. In addition to the large number of genes that likely contribute to these disorders, environmental factors, as well as interactions between genes or between genes and environmental factors, almost certainly also play a role. Indeed, several causal pathways may lead to either disorder or to both. Despite the complexity of these disorders, some findings regarding candidate genes have been replicated across studies and therefore appear robust. However, as advances in genetic technology have resulted in refined gene mapping techniques, it is equally important to identify refined phenotypes that distinguish the various pathways that lead to alcohol and nicotine dependence. The combination of clear distinctions among disease processes at the phenotypic level and the recent explosion in genetic technologies will dramatically increase researchers’ abilities to identify additional genetic factors as well as gene–environment interactions that contribute to alcohol and nicotine dependence.

**At a Glance**

**The Collaborative Study on the Genetics of Alcoholism (COGA)**

- COGA is a comprehensive family and genetic study of alcohol dependence and related disorders.
- COGA researchers collect data on genetic and biologic factors related to alcohol use, abuse, and dependence as well as on behavioral variables, psychiatric disorders, personality traits, family history, and other risk factors, including smoking behavior.
- The study includes patients seeking treatment for alcoholism (probands) and their families.
- Families in which the proband and at least two siblings are alcohol dependent are used for genetic studies.
- Researchers determine the influence of genetic factors on development of alcohol dependence and/or habitual smoking using family studies, genetic linkage analyses, and candidate gene association studies.
- In family studies of COGA families, siblings of an alcohol-dependent proband were at 1.7 times higher risk of becoming habitual smokers than were siblings of people who were not alcohol dependent. Habitual smoking in alcohol-dependent probands further increased their siblings’ risk of becoming habitual smokers.

Genetic studies are uniquely capable of providing insight into the biological pathways involved in the development of alcohol and nicotine dependence because ethical considerations preclude many experimental research designs involving human subjects. The newest generation of human genetic studies exploits the copious natural variability in the genome to evaluate effects of specific gene variations on disease outcomes. Identification of these genes provides important first clues to the biological underpinnings of disease. Although the complexity of alcohol and nicotine dependence makes the search for genes more difficult, it also underscores the need for genetic studies of these disorders. If various developmental pathways can lead to these common addictions, it is unlikely that any single treatment or prevention approach will be optimal for all vulnerable people. Because human genetic studies can uncover diverse biological factors involved in these disorders, such approaches have great potential for alleviating the enormous public health burden stemming from alcohol and nicotine dependence.

## Footnotes

1 The term “phenotype” refers to the observable characteristics of an individual, such as color of the eyes or presence of a disorder. Conversely, the term “genotype” refers to the individual’s genetic makeup—that is, the specific gene variants determining the phenotype in that individual.

2 Acetylcholine also interacts with another receptor, the nicotinic receptor. As the name implies, this also is the receptor with which nicotine interacts in order to exert its effects on the brain.

## References

[b1-172-178] American Psychiatric Association (APA) (1987). Diagnostic and Statistical Manual of Mental Disorders, Third Edition, Revised.

[b2-172-178] Bierut LJ, Dinwiddie SH, Begleiter H (1998). Familial transmission of substance dependence: Alcohol, marijuana, cocaine, and habitual smoking. A report from the Collaborative Study on the Genetics of Alcoholism. Archives of General Psychiatry.

[b3-172-178] Bierut LJ, Rice JP, Goate A (2004). A genomic scan for habitual smoking in families of alcoholics: Common and specific genetic factors in substance dependence. American Journal of Medical Genetics Part A.

[b4-172-178] Bierut LJ, Schuckit MA, Hesselbrock V, Reich T (2000). Co-occurring risk factors for alcohol dependence and habitual smoking. Alcohol Research & Health.

[b5-172-178] Bymaster FP, Felder CC (2002). Role of the cholinergic muscarinic system in bipolar disorder and related mechanism of action of antipsychotic agents. Molecular Psychiatry.

[b6-172-178] Cousins MS, Roberts DC, de Wit H (2002). GABA(B) receptor agonists for the treatment of drug addiction: A review of recent findings. Drug and Alcohol Dependence.

[b7-172-178] Covault J, Gelernter J, Hesselbrock V, Nellissery M, Kranzler HR (2004). Allelic and haplotypic association of GABRA2 with alcohol dependence. American Journal of Medical Genetics Part B: Neuropsychiatric Genetics.

[b8-172-178] Davies M (2003). The role of GABAA receptors in mediating the effects of alcohol in the central nervous system. Journal of Psychiatry and Neuroscience.

[b9-172-178] Dick DM, Bierut LJ (2006). The genetics of alcohol dependence. Current Psychiatry Reports.

[b10-172-178] Dick DM, Foroud T (2003). Candidate genes for alcohol dependence: A review of genetic evidence from human studies. Alcoholism: Clinical and Experimental Research.

[b11-172-178] Edenberg HJ, Dick DM, Xuei X (2004). Variations in GABRA2, encoding the alpha 2 subunit of the GABA(A) receptor, are associated with alcohol dependence and with brain oscillations. American Journal of Human Genetics.

[b12-172-178] Edenberg HJ, Xuei X, Chen HJ (2006). Association of alcohol dehydrogenase genes with alcohol dependence: A comprehensive analysis. Human Molecular Genetics.

[b13-172-178] Feighner JP, Robins E, Guze SB (1972). Diagnostic criteria for use in psychiatric research. Archives of General Psychiatry.

[b14-172-178] Fehr C, Sander T, Tadic A (2006). Confirmation of association of the GABRA2 gene with alcohol dependence by subtype-specific analysis. Psychiatric Genetics.

[b15-172-178] Gelernter J, Liu X, Hesselbrock V (2004). Results of a genomewide linkage scan: Support for chromosomes 9 and 11 loci increasing risk for cigarette smoking. American Journal of Medical Genetics Part B: Neuropsychiatric Genetics.

[b16-172-178] Grant BF, Hasin DS, Chou SP, Stinson FS, Dawson DA (2004). Nicotine dependence and psychiatric disorders in the United States: Results from the National Epidemiologic Survey on Alcohol and Related Conditions. Archives of General Psychiatry.

[b17-172-178] Harwood H (2000). Updating Estimates of the Economic Costs of Alcohol Abuse in the United States: Estimates, Update Methods, and Data.

[b18-172-178] Hemrick-Luecke SK, Bymaster FP, Evans DC, Wess J, Felder CC (2002). Muscarinic agonist-mediated increases in serum corticosterone levels are abolished in m(2) muscarinic acetylcholine receptor knockout mice. Journal of Pharmacology and Experimental Therapeutics.

[b19-172-178] Iacono WG, Carlson SR, Malone SM, McGue M (2002). P3 event-related potential amplitude and the risk for disinhibitory disorders in adolescent boys. Archives of General Psychiatry.

[b20-172-178] Jones KA, Porjesz B, Almasy L (2004). Linkage and linkage disequilibrium of evoked EEG oscillations with CHRM2 receptor gene polymorphisms: Implications for human brain dynamics and cognition. International Journal of Psychophysiology.

[b21-172-178] Lappalainen J, Krupitsky E, Remizov M (2005). Association between alcoholism and gammaamino butyric acid alpha2 receptor subtype in a Russian population. Alcoholism: Clinical and Experimental Research.

[b22-172-178] Li MD (2006). The genetics of nicotine dependence. Current Psychiatry Reports.

[b23-172-178] Li MD, Ma JZ, Cheng R (2003). A genomewide scan to identify loci for smoking rate in the Framingham Heart Study population. BMC Genetics.

[b24-172-178] Luo X, Kranzler HR, Zuo L (2005). CHRM2 gene predisposes to alcohol dependence, drug dependence and affective disorders: Results from an extended case-control structured association study. Human Molecular Genetics.

[b25-172-178] Mokdad AH, Marks JS, Stroup DF, Gerberding JL (2004). Actual causes of death in the United States, 2000. Journal of the American Medical Association: JAMA.

[b26-172-178] Porjesz B, Almasy L, Edenberg HJ (2002). Linkage disequilibrium between the beta frequency of the human EEG and a GABAA receptor gene locus. Proceedings of the National Academy of Sciences of the USA.

[b27-172-178] Porjesz B, Begleiter H (2003). Alcoholism and human electrophysiology. Alcohol Research & Health.

[b28-172-178] True WR, Xian H, Scherrer JF (1999). Common genetic vulnerability for nicotine and alcohol dependence in men. Archives of General Psychiatry.

[b29-172-178] Tyndale RF (2003). Genetics of alcohol and tobacco use in humans. Annals of Medicine.

[b30-172-178] Wang JC, Hinrichs AL, Stock H (2004). Evidence of common and specific genetic effects: Association of the muscarinic acetylcholine receptor M2 (CHRM2) gene with alcohol dependence and major depressive syndrome. Human Molecular Genetics.

